# Synthesis and Characterization of Copoly(Ether Sulfone)s with Different Percentages of Diphenolic Acid Units

**DOI:** 10.3390/polym12081817

**Published:** 2020-08-13

**Authors:** Andrea A. Scamporrino, Concetto Puglisi, Angela Spina, Maurizio Montaudo, Daniela C. Zampino, Gianluca Cicala, Giulia Ognibene, Chiara Di Mauro, Sandro Dattilo, Emanuele F. Mirabella, Giuseppe Recca, Filippo Samperi

**Affiliations:** 1 Institute for Polymers Composites and Biomaterials, IPCB-SS Catania CNR, Via Paolo Gaifami 18, 95126 Catania, Italy; concetto.puglisi@cnr.it (C.P.); angelaspina1@virgilio.it (A.S.); mmontaud@unict.it (M.M.); danielaclotilde.zampino@cnr.it (D.C.Z.); sandro.dattilo@cnr.it (S.D.); emanuelefrancesco.mirabella@cnr.it (E.F.M.); giuseppe.recca@gmail.com (G.R.); filippo.samperi@cnr.it (F.S.); 2 Department of Civil Engineering and Architecture, University of Catania, Viale Andrea Doria 6, 95125 Catania, Italy; gcicala@unict.it (G.C.); giuliaognibene@live.com (G.O.); chiara.dimauro@hotmail.it (C.D.M.)

**Keywords:** synthesis, random copolymers, poly (ether sulfone)s, diphenolic acid, polyelectrolytes, NMR, DMA

## Abstract

New functionalized Poly(ether sulfone)s having different molar ratio (10, 20, 30, 50, 70, 100 mol%) of 4,4-bis phenoxy pentanoic acid unit (diphenolic acid; DPA) units were synthesized and characterized by (^1^H and ^13^C)-NMR, MALDI-TOF MS, FT-IR, DSC and DMA analyses. The microstructural analysis of the copolymers, obtained by ^13^C-NMR using an appropriate statistical model, shows a random distribution of copolymer sequences, as expected. The presence of different amount of DPA units along the polymer chains affects the chemical and physical properties of the copolymers. The Tg and the contact angle values decrease as the molar fraction of DPA units increases, whereas the hydrophilicity increases. NMR and MALDI-TOF MS analyses show that all polymer chains are almost terminated with hydroxyl and chlorine as end groups. The presence of cyclic oligomers was also observed by MALDI-TOF MS analysis.

## 1. Introduction

**Scheme 1:**
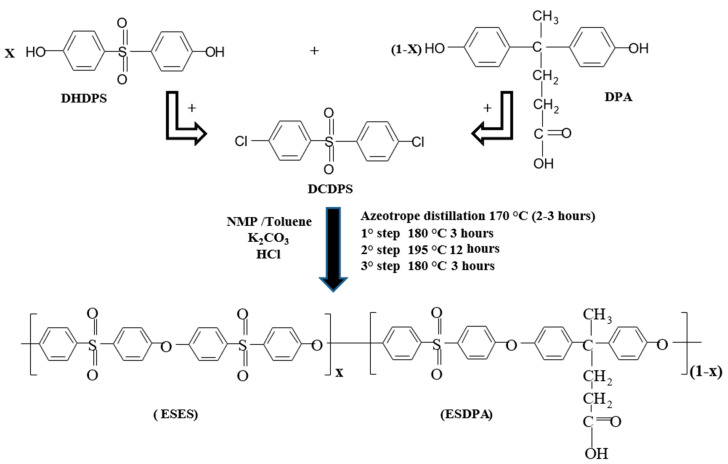
Schematic picture of P(ESES-co-ESDPA) copolymer synthesis.

Engineering thermoplastic aromatic poly(ether sulfone)s (PES)s show remarkable chemical and thermal stability, excellent strength and flexibility, transparency, as well as high glass transition temperature, good film forming properties, resistance to hydrolysis and oxidation processes, good optical properties, resistance to extreme pH values, and low creep [1,2,3,4,5,6,7,8,9,10,11,12,13,14,15,16]. As a function of their inherent properties, (PES)s have found remarkable application in the fields of materials science, biology and polymer science [1,2,3,4,5,6,7,8,9,10,11,12,13,14,15,16,17,18,19,20,21,22,23,24,25,26,27,28,29,30,31]. However, despite the remarkable progress in their synthesis and applications, the materials have some limitations, including stress cracking with certain solvents, poor resistance to cracking and weathering. The introduction of functional groups into the polysulfone main chains could overcome these limitations and also extend the range of potential applications of these polymers through the specific properties gained. The literature reports that a wide range of chemical, mechanical, physical, morphological and thermal characteristics can be obtained by functionalization of the made PESs or varying the structure of the monomer units in the copolymers synthesized [16,17,18,19,20,21,22,23,24,25,26]. Due to their antimicrobial action, solubility and separation characteristics, water permeability, the functional groups, which modify the hydrophilicity of polysulfones, are of particular interest for biomedical applications, and in general, the material sciences [16]. Furthermore, the functional groups are an intrinsic requirement for affinity, ion exchange, and other special membrane properties. The introduction of specific functionality to PES has been accomplished by: (I) The functional monomer approach allowing modification at the polymerization stage; (II) the post-functionalization of commercial polymers [3,6,10,17,18,19,20,26,28,30,31]. The different functional groups linked to the PES chains include acids, alcohols, amine, halides, phenyl and polymerizable groups [3,6,10,17,18,19,20,26,28,30,31,32]. Dizman et al. (2013) have discussed specific examples of both functional monomer and post functionalization methods [4]. Several authors have introduced the hydrophilic carboxylic (COOH) function into PES to overcome the problems due to its hydrophobicity [3,17,18,19,20,26,28,30,31,32]. COOH groups not only induce/lead to hydrophilicity, but also increase the pH sensitivity [32], as well as the blood compatibility of PES materials [33]. The need for thermally stable polymeric materials has suggested the possibility of using polyethersulfones with specific ion-conducting site such as COOH groups in the backbone. In order to obtain functionalized co-poly(arylene ether sulfone)s with active carboxylic acid (COOH) pendant groups, in the present work some random copolymers were synthesized by solution polyetherification carried out by nucleophilic displacement polycondensation reaction between stoichiometric amount of 4,4’-dichlorodiphenyl sulfone and different molar ratio of 4,4′dihydroxydiphenylsulfone (DHDPS) and 4,4-bis(4-hydroxyphenyl)pentanoic acid (diphenolic acid, DPA), as sketched in Scheme 1. DPA was chosen for its structural resemblance to bisphenol A, which is used in the synthesis of polysulfones. Its polycondensation with dihalogenated diphenylsulfone has been thoroughly studied [17,18]. Moreover, the presence of COOH groups can permit to prepare membranes for fuel cells and also antibacterial membranes, using these polymers as ionic liquids coupling with appropriate cations. The synthesized random copolymers are here referred as P(ESES-co-ESDPA). The chemical structural characterization of all polymers and copolymers was performed by FT-IR, (^1^H, ^13^C)-NMR, and MALDI-TOF MS analysis. Detailed information on the chemical structures and composition of the P(ESES-co-ESDPA) samples was obtained by (^1^H, ^13^C)-NMR analyses applying appropriate Bernoullian statistical models. The thermal properties of the whole set of copolymers were studied by DSC and DMA tools and compared with those of the corresponding homopolymers. Furthermore, the hydrophilic behavior as a function of the ES-DPA molar ratio was evaluated by static contact angle (SCA) measurements. Preliminary results on capability of the P(ESES-co-ESDPA) based membrane to remove heavy metals from water solution were also showed.

## 2. Experimental

### 2.1. Materials

4,4^1^-dicholrodiphenylsulfone (DCDPS), 4,4^1^dihydroxydiphenylsulfone (DHDPS), 4,4-Bis(4-hydroxyphenyl) pentanoic acid (DPA, diphenolic acid), potassium carbonate (K_2_CO_3_), N-Methyl-2-pyrrolidone (NMP), toluene, methanol, trans-2-[3-(4-tert-Butylphenyl)-2-methyl-2-propenylidene]malononitrile (DCTB), 3-indoleacrylic acid (IAA), α-Cyano-4-hydroxycinnamic acid (α-Cyano), tetrahydrofuran (THF) and Dimethyl sulfoxide-d_6_ (DMSO-d6) were purchased by Aldrich (Milan, Italy). DCDPS, DHDPS, and DPA were dried at 80 °C under vacuum before their use; K_2_CO_3_ was dried at 150 °C under vacuum, whereas the other products were used without any other treatment.

### 2.2. Synthesis

Copolymers with different molar composition in ESES (ether sulfone ether sulfone) and ESDPA (ether sulfone diphenolic acid sulfone) units were synthesized via nucleophilic condensation of DCDPS with stoichiometric amounts of DHDPS and DPA, as sketched in Scheme 1. The molar ratio of DHDPS and DPA monomers was varied (100:0; 90:10; 80:20; 70:30; 50:50; 30:70: 0:100), in order to synthesize copolymers with different molar composition. Their corresponding homopolymers were also synthesized at the same experimental conditions. As example, to prepare the copolymer P(ESES-co-ESDPA) 50:50, equimolar amounts of DHDPS (10.89 g, 43.5 mmol) and DPA (12.45 g, 43.5 mmol) were solubilised in NMP (ratio 1:1.5 *w/v*) in a three necks round flask equipped with a magnetic stirrer, a dropping funnel, a nitrogen inlet and a reflux condenser. After that, 40 mL of toluene and a solution of K_2_CO_3_ (13.24 g, 95.8 mmol) in 50 mL of NMP were added and the mixture was heated, under stirring, at 170 °C until the water/toluene azeotrope distilled. In this way it is possible to definitively remove the water inside the reaction ambient. Thereafter, an anhydrous NMP solution (50 mL) of DCDPS (25.0 g, 87.0 mmol) was added to the reaction mixture and the temperature was raised and maintained at 180 °C for 3 h. During this reaction time the solution has took on a dark amber colour. Then, the reaction temperature was raised up to 195 °C and maintained at this temperature for 12 h; successively the temperature was lowered to 180 °C and the reaction was carried out for further 3 h. After that, the reaction mixture was cooled down to the room temperature and 10 mL of water solution of HCl (12 M) were added under stirring to protonate the pendant carboxyl groups. So, the polymer solution was added, drop by drop, into a 50:50 *v/v* water/methanol mixture (1.5 L), in order to obtain a fine light amber precipitate. The solution was filtered on a Buchner and then washed several times with cold methanol, finally the powder was dried at 110 °C under vacuum (10 Torr) overnight. To remove the low molar mass (MM) oligomers, the crude polymers were further purified by continuous extraction in a Soxhlet apparatus. THF, for either P(ESES) homopolymer and P(ESES-co-ESDPA) copolymers with a molar composition of ESDPA units from 10 up to 30 mol%, and ethyl ether, for the copolymers with an ESPDA molar composition higher than 30%, since these ones are not soluble in THF, were used. Purified copolymers were characterized by FT-IR, (^1^H and ^13^C)-NMR and MALDI-TOF MS methods. Either the P(ESES-co-ESDPA) copolymers 50:50 and 30:70, and the P(ESDPA homopolymers were analysed by SEC being soluble in THF. All extracted oligomers were characterized by MALDI-TOF MS and SEC analyses.

### 2.3. Methods of Analysis

#### 2.3.1. H-NMR and ^13^C-NMR

^1^H-NMR and ^13^C-NMR spectra of the whole set of synthesized homo and copolymers were obtained by a Bruker Advance 400 spectrometer (Milan, Italy). The analyses were performed at 50 °C at 40,013 MHz for protons and at 100 MHz for carbon analyses. Samples were dissolved in deuterated dimethyl sulfoxide (DMSO-d_6_) at the concentration of 25 mg/mL. The chemical shifts and the assignments of the protons and carbons belonging to the polymer samples are summarized in Table 2.

#### 2.3.2. MALDI-TOF MS

MALDI-TOF mass spectra of all polymers were recorded by a 4800 Proteomic Analyzer (Applied Biosystems, MA, USA) MALDI-TOF/TOF instrument equipped with a Nd:YAG laser at a wavelength of 355 nm with <500 ps pulse and 200 Hz firing rate, operating in reflectron mode. The accelerating voltage was 15 kV. External calibration was performed using an Applied Biosystems calibration mixture consisting of polypeptides with different molar mass values. The irradiance was maintained slightly above the threshold to obtain a mass resolution of about 3000–5000 fwhm. Mass accuracy was about 50 ppm. Several matrices: 1,8,9-anthracenetriol (dithranol); α-Cyano-4-hydroxycinnamic acid (CHCA); trans-2-[3-(4-tert-butylphenyl)-2-methyl-2-propenylidene]malononitrile (DCTB); trans-3-indoleacrylic acid (IAA)) were used to analyze the polymers samples synthetized. In the present work are reported the best mass spectra recorded using DCTB and IAA as matrices.

#### 2.3.3. FT-IR

The FT-IR spectra were recorded by a Perkin-Elmer Instruments (Milan, Italy), the Spectrum One FT-IR Spectrometer having a lithium tantalate (LiTaO_3_) single crystal detector. Polymer samples were analyzed as thin films obtained by electrospinning method. The spectra were acquired in the Middle Infrared (4000–400 cm^−1^) in modality ATR and processed through the software Spectrum One.

#### 2.3.4. Differential Scanning Calorimetry

Each sample was investigated about its glass transition temperature (Tg) by the TA Instruments Q100 DSC calorimeter. The instrument was calibrated evaluating the melt purity of an Indium standard (156.6 °C and 28.45 J/g), as reported in the user manual provided by the producer.

#### 2.3.5. Dinamo Mechanical Analysis (DMA)

DMA analysis was carried out on a dynamic mechanical thermal analyser (TRITEC2000, Triton Technology, Leicestershire, UK) by single cantilever geometry. P(ESES-co-ESDPA) copolymers were tested using the pocket DMA (Leicestershire, UK)) approach in their powder form. The pocket DMA is a technique used in the pharmaceutical field [34] and for polymer blends [35] for testing powders. The polymers, obtained from the syntheses, were finely micronized in powder with 30 µm average size. Then, 0.35 g of polymer powder, previously dried at 80 °C overnight, was weighted in a standard stainless-steel pocket purchased from Triton and pressed to obtain a uniform thickness of 0.8 mm. The test was carried out accordingly to the following protocol. The sample was stabilized at 25 °C and then heated up to 290 °C at 5 °C/min. After this first scan, samples were cooled down at room temperature and again heated up to 290 °C at 5 °C/min. Similar techniques were also reported by Carlier et al. [36] for organic polymers under the name “supported DMA”. This technique allows the direct evaluation of thermal transitions from E’ and tan δ traces. However, the absolute values of E’ and tan δ for the polymer are influenced by the presence of the metal pocket and thus, the real values should be analysed out considering the assembly as a sandwich material. The tan δ versus temperature was plotted for the sample after the second scan.

#### 2.3.6. Size Exclusion Chromatography (SEC)

The SEC measurements of the polymer sample soluble in THF were carried out by a Waters 515 apparatus (Milan, Italy ), equipped with four Ultrastyragel HR columns (ID = 7.8 mm, L = 300 mm, 5 µm of particle size) in the order HR-4, HR-3, HR-2 and HR-1 connected in series and a Waters R401 detector, using THF as mobile phase (1 mL/min of flow rate). The SEC traces were processed using a Clarity-GPC software (Data Apex, Prague, Czech Republic) provided by DataApex and applying the calibration curve built using PS narrow standards.

#### 2.3.7. Wettability Analysis

Contact angle measurements and drop shape analysis were carried out on the electrospun fibers realized with the synthesized copolymers. The measurements were performed by an OCA 15 EC Optical Contact Angle meter, with the support of the SCA software by DataPhysics Instruments GmbH (San Jose, CA, USA).

#### 2.3.8. Viscosimetry

The inherent viscosity (η_inh_ = lnη_rel_/C; C = 0.5 g/dL) was determined by using a Ubbelohde suspended-level viscometer in dimethyl formamide (DMF) as solvent at 40 °C. The measured values are reported in Table 1.

#### 2.3.9. Chemical Microstructural Analysis and Sequence Distribution of P(ESES-co-ESDPA) Copolymers

In order to calculate compositions and microstructures of copolymers we used ^1^H-NMR and ^13^C-NMR data applying an appropriate chemical microstructural model in accord to the literature [8,37,38,39,40,41,42]. Experimental degree of polymerization (X_n_) and the number average molar mass (Mn) of each copolymer was calculated from the integrated area of the peaks due to the aromatic protons in ortho to the phenol chain ends (protons H_2′_ and H_10′_) via comparison with peaks, due aromatic protons in ortho position to the ether-oxygen groups in the backbone (protons H_10_, H_2_ and H_7_) and in ortho to the phenyl-pentanoic group of the DPA units (H_11_), using the following Equations (1)–(3).

Equation (1)

(1) Xn=I10+2+I11+78I2′+10′4

Equation (2)

(2) Mr.u.=CESES×464.4gmol+CESDPA×500.4gmol

where 464.4 and 500.4 are the mass of the ESES, and ESDPA repeat units, respectively.

Equation (3):

(3) $$M_{n}=\left(X_{n}+1\right)\cdot M_{r.u.}$$

The molar composition was calculated from the ^1^H-NMR spectra using the integrated area of aromatic protons H_10_, H_2_, H_7_ and H_7_, applying the Equations (4) and (5). The molar composition was also determined from ^13^C-NMR spectra using the intensity of the quaternary carbons C1 and C8.

Equation (4):

(4) $$C_{\frac{ESES}{ESDPA}=\frac{I_{10+2}/12}{I_{11+7}/8}}$$

Equation (5)

(5) $$C_{ESES}+C_{ESDPA}=1$$

The molar composition was also determined from ^13^C-NMR spectra using the intensity of the quaternary carbons C1 and C8 which resonate around 159.2, and 161.2 ppm, respectively (see Table 2), using the Equations (6) and (7):

Equation (6):

(6) $$C_{ESES}=\frac{I_{159.2}+I_{159.25}}{I_{159.2}+I_{159.25}+I_{159.03}+I_{161.25}+I_{161.09}}.$$

Equation (7)

(7) $$C_{ESDPA}=\frac{I_{161.25}+I_{161.09}+I_{159.03}}{I_{159.2}+I_{159.25}+I_{159.03}+I_{161.25}+I_{161.09}}.$$

**Scheme 2:**
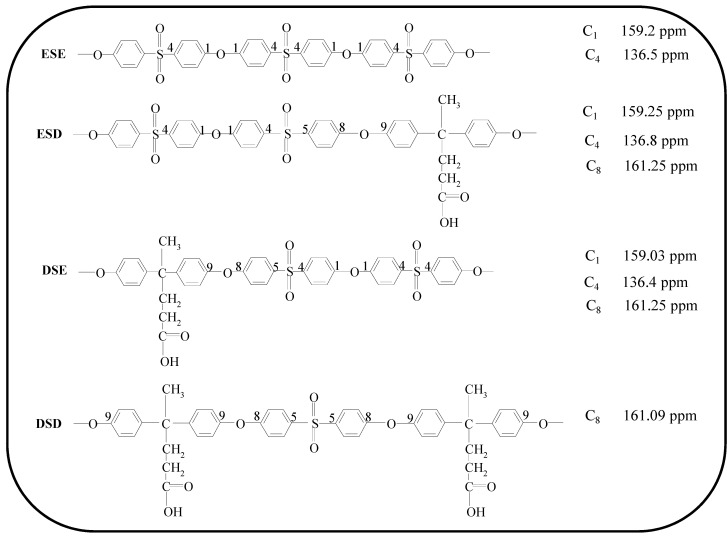
Structures of the triads sequences centred on diphenyl sulfone (S) moiety, and the chemical shifts of their carbons C1 and C8.

The molar fraction of triads sequences centered on diphenylsulfone moiety (S) as ESE, ESD, DSE, DSD (see Scheme 2) were estimated from ^13^C-NMR spectra applying the Equations (8)–(11), using the normalized intensity of the quaternary carbons C1 and C8 (Table 2) linked to the oxygen of the ether groups in the in these equations D indicate the DPA moiety.

The chemical microstructure of the copolymers such as the sequence distribution of the dyads and triads, their average sequence length and the degree of randomness (β) of the P(ESES-co-ESDPA) copolymers were also calculated from ^13^C-NMR spectra applying the Equations (8)–(11). The intensity of the quaternary carbons linked to the oxygen of the ether groups in the backbone belonging to the triad sequences centered on diphenylsulfone moiety (S) as E-S-E, E-S-DPA, DPA-S-E, DPA-S-DPA (see Scheme 2), have been used. The quaternary Carbons C1 belonging to the triad sequences E-S-E, E-S-DPA, DPA-S-E resonate at 159.2 ppm, 159.25 ppm and 159.03 ppm, respectively, the C8 ones resonate at 161.25 ppm (triads E-S-DPA and DPA-S-E) and at 161.09 ppm (triad DPA-S-DPA), respectively. The results are showed in Table 3:

Equation (8)

(8) fE−S−E=I159.22I159.22+I159.25+I159.03+I161.25+I161.09+0.015.

Equation (9)

(9) fE−S−DPA=23(I159.25+I161.25)I159.22+I159.25+I159.03+I161.25+I161.09+0.015.

Equation (10)

(10) fDPA−S−E=23(I159.03+I161.25)I159.22+I159.25+I159.03+I161.25+I161.09+0.015

Equation (11)

(11) fDPA−S−DPA=I161.09I159.22+I159.25+I159.03+I161.25+I161.09+0.015

Taking into account that the E and DPA moieties must be linked only to the S unit alone and does not together, the probability (P_E-DPA_ and P_DPA-E_) of finding the E (or DPA) unit next to other the DPA (or E) one units can be calculated from the triad molar fractions applying the Equations (12) and (13), in accord to the literature [40,41]:

Equation (12)

(12) $$P_{E-DPA}=\frac{f_{E-S-DPA}}{2\left(f_{E-S-E}+\frac{f_{E-S-DPA}}{2}\right)}$$

Equation (13)

(13) $$P_{DPA-E}=\frac{f_{ESD}}{2\left(f_{DPA-S-DPA}+\frac{f_{E-S-DPA}}{2}\right)}$$

The number-average sequential length of the ESES (L_ESES_) and ESDPA (L_ESDPA_) repeat units are calculated by the following Equations (14) and (15):

Equation (14)

(14) $$L_{ESES}=\frac{1}{P_{E-DPA}}$$

Equation (15)

(15) $$L_{ESDPA}=\frac{1}{P_{DPA-E}}$$

The degree of randomness (β) is defined by Equation (16)

Equation (16)

β = P_E-DPA_ + P_DPA-E_ (16)

## 3. Results and Discussion

### 3.1. Infrared Spectroscopy

**Figure 1:**
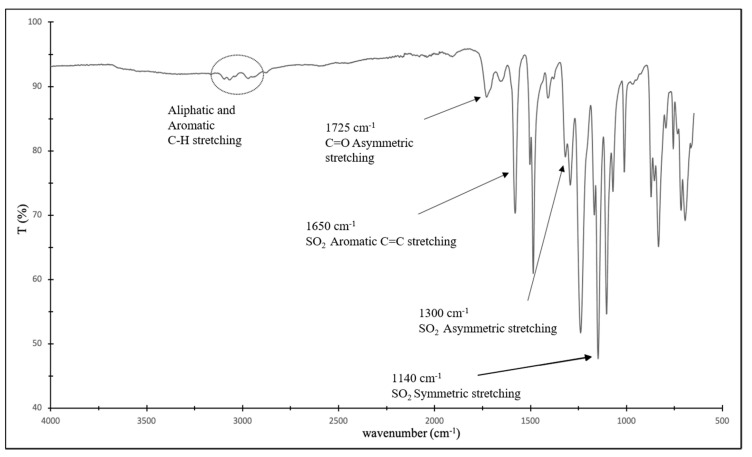
FTIR spectrum of the P(ESES-co-ESDPA) 30:70 copolymer.

Co-poly(ether sulfone)s bearing active carboxyl acid moiety along the chains (P(ESES-co-ESDPA)) were synthesized via aromatic nucleophilic substitution polymerization of stoichiometric amount of DCDPS with different molar ratio of DHDPS and DPA; the polycondensation was carried out at high temperature in NMP/toluene azeotrope in presence of anhydrous K_2_CO_3_, as schematized in Scheme 1. The corresponding homopolymers were also synthesized in the same conditions. The reaction of DCPS with DHDPS generates the alternate sequences ether sulfone-ether sulfone (ESES), whereas the one with DPA produces the functionalized sequences ESDPA, as highlighted in Scheme 1. The chemical structure and composition of all copolymers were investigated by means of FT-IR and (^1^H and^13^C)-NMR methods. Figure 1 shows the FTIR spectrum of the 30:70 copolymer film, recorded in modality ATR. It shows the typical absorption peaks due to; the stretching of aromatic CH in the range 3020–3095 cm^−1^; the C-H asymmetric and symmetric stretching of -CH_2_ groups in the range 2850–3000 cm^−1^; the stretching of C=C of aromatic rings at 1650 cm^−1^; the asymmetric stretching of the SO_2_ bond in the range 1350–1275 cm^−1^; the symmetrical stretching of SO_2_ bond in the range 1175–1125 cm^−1^; the asymmetric stretching of C=O bond of the -COOH groups at 1725 cm^−1^. In the supporting information (Figure S1) is reported the overlay of the spectra, which shows the formation of the P(ESES-co-ESDPA) copolymers. In particular, the relative intensity of the diagnostic absorption peaks due to the COOH groups at 1725 cm^−1^ increases with respect to that of the SO_2_ moieties as raises the molar amount of the DPA units in the copolymer chains. Figure S1 show FT-IR spectra of all copolymers. The adsorption bands at about 1347 cm^−1^ and 1140 cm^−1^ due to the asymmetric and symmetric stretching SO_2_ moiety, respectively, were assigned in accord to the literature [17,20,27,28,29].

### 3.2. NMR Analysis

**Figure 2:**
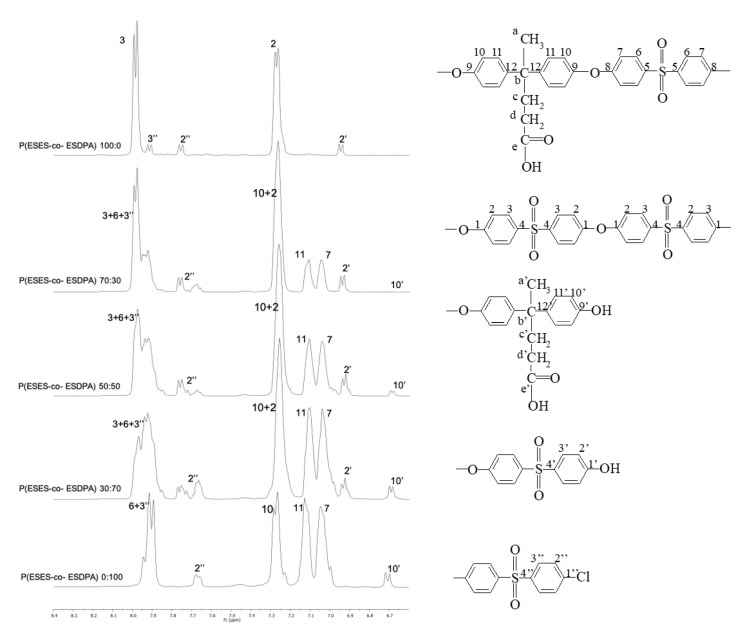
^1^H-NMR spectra in the aromatic region between 6.4 and 8.4 ppm of five polymer samples.

To obtain more information on the chemical composition and on the microstructure, all copolymers were investigated by (^1^H and ^13^C)-NMR, using deuterated DMSO as solvent. The spectra were recorded at 50 °C to a better resolution. The chemical shifts resonances were accurately assigned to the specific protons and carbons, according to published data [8] and to the corresponding homopolymers ones. The specific assignments are reported in Table 2. As an example, the (^1^H and ^13^C)-NMR spectra of the P(ESES-co-ESDPA) 50:50 copolymer are depicted in Figure S2 together with the specific assignments. The aromatic protons resonate in the range between 6.5 and 8.2 ppm, whereas the aliphatic protons of the DPA units resonate between 1.5 and 2.5 ppm. The methyl groups (H_a_) give a singlet a 1.59 ppm, whereas the protons of the methylene in α (H_d_) and in β (H_c_) to the carboxylic acid resonate at 2.11 and 2.38 ppm, respectively. The ^1^H-NMR spectra of all copolymers as well that of the P(ESDPA) homopolymer show a large signal at 12.09 ppm, due to the protons (H_e_) of the pendant carboxylic groups. The spectra show also the peaks (singlet) at 10.65 and 9.26 ppm due to resonance of phenol (OH) end groups belonging to the DHDPS, and DPA end groups, respectively (see Table 2). The aromatic protons in ortho to the OH-phenols end chains belonging to the DHDPS and DPA end chains resonate at 6.95 ppm (d, H_2′_) and 6,69 ppm (d, H_10′_), respectively. The unresolved doublet at about 7.68 ppm is assigned to aromatic protons in ortho to the chlorine belonging to the DCDPS end chains (protons H_2″_). Therefore, the ^1^H-NMR analysis gave pertinent information on the end groups of the copolymer chains. Figure 2 shows the ^1^H-NMR spectra in the aromatic regions ranging from 6.6 to 8.6 ppm of some polymer samples. As an example, the total ^1^H-NMR spectrum of the P(ESES-co-ESDPA) 50:50 sample is reported in the Figure S2a. The molar amounts, relative to the feed, of ESES and ESDPA units in the copolymers were calculated from proton NMR spectra using the integrated areas of the peaks due to the aromatic protons which resonate in the range 6.9–8.1 ppm, applying the Equations (4) and (5). The results are noted in Table 1. The calculated molar composition of all samples agrees with that from the feed. Some differences can be due to the low resolution of the aromatic protons. Polymer samples with a Mn of about 6000 g/mol were obtained in the experimental conditions applied, as shown in Table 1. Nevertheless, due to the lack of peak separation in the aromatic regions and to their complexity, it was not possible to unequivocally assign each peak to the corresponding sequences (dyads, triads, etc.) in the copolymer chains by the proton spectra. To overcome this problem, all samples were analysed by ^13^C-NMR, in order to define all structural units along their chains. Moreover, we will point out the merits of sequence analysis for the characterization of molar compositions and microstructure of the copolymers synthesized changed the DHDPS/DPA molar ratio in the feed. The knowledge of these parameters could allow in the further studies to correlate copolymers properties (thermal, mechanical, membrane for separating gases, selective extraction of heavy metals, etc.) to their compositions or to obtain/gather/deduct information on (to foresee) copolymers properties from their compositions.

**Figure 3:**
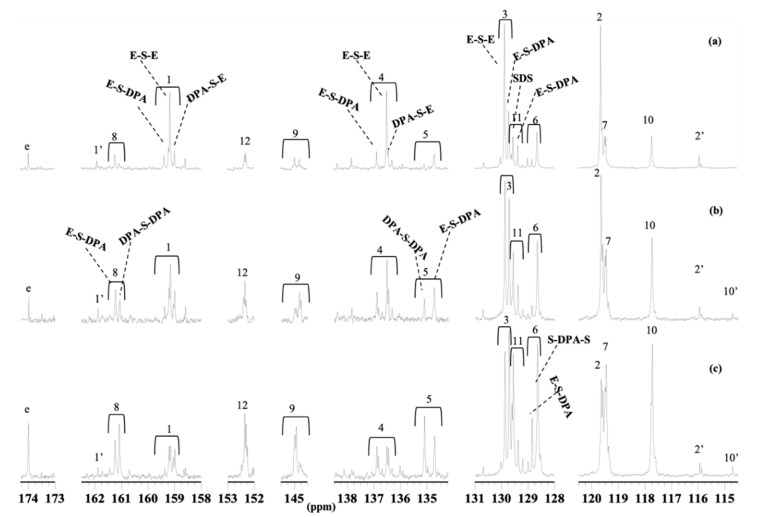
Enlarged section of seven ranges with assignments of the described spectra.

The aromatic quaternary carbons are more sensitive to the sequence effects of aryl ether copolymers than any other aromatic carbons, due to the occurrence of through-space and through-bond interactions between neighboring units [8,38,39,41]. In many cases the aromatic quaternary carbons linked to the ether-oxygen, as well the ones linked to the sulfone group are extremely sensitive to the sequence distributions along the chains, since their assignments, either to the corresponding dyads and triads sequences, are often unequivocally possible. Therefore, in many cases they give fundamental sequencing information [8,40]. Figure 3 shows the expanded ^13^C-NMR spectra of the P(ESES-co-ESDPA) copolymers 70:30, 50:50 and 30:70, in the aromatic regions between 114.5 and 174.3 ppm. The chemical shifts were accurately assigned to the specific carbons on the basis of those of homopolymers (P(ESES) and P(ESDPA) (Table 2). The ^13^C-NMR spectra in Figure 3 show relevant changes in the relative intensity of quaternary carbons C1 and C8 belonging to the phenyl ether moieties belonging to of the phenylether-phenylsulfone (ES) and phenylsulfone-DPA (ESDPA) units, respectively (see Table 2 and Scheme 2), which resonate in the range 158.8–161.3 ppm. The relative intensity of the signals C8 and C1 changes as a function of the ESES/ESDPA molar ratio in the copolymers. Likely, relevant changes were also observed for the quaternary carbons linked to the sulfone groups (C4 and C5; Table 2 and Scheme 2), which resonate in the range between 143.5 and 137.2 ppm. Distinct differences were also observed either for the signals due to the tertiary carbons in ortho to the sulfone groups (C3 and C6) and to the tertiary carbons in ortho to the ether-oxygen groups (C2 and C7), as changes the molar composition of the copolymers. As expected, the aromatic tertiary carbons of the DPA units (C10 and C11) do not show relevant multiplicity of peaks, due to the sequence distribution, because the aliphatic pentanoic acid moiety hampers the bond interactions between neighboring units. Their relative intensity changes with respect to that of the homologous carbons belonging to the ES sequences (i.e., carbons 2, 3, 6 and 7), as a function of the molar composition. The aliphatic region of ^13^C-NMR spectra of the copolymer samples is showed in Figure S4, whereas that of the homopolymers is displayed in Figures S5 and S6. The good resolution of the peaks due to the quaternary carbons C_1_, C_8_, C_4_ and C_5_ has permitted their assignments to the triad sequences centred on the diphenyl sulfone units (S) present along the copolymer chains. The pertinent assignments are reported in Figure 3 and the corresponding structures are depicted in Scheme 2. In fact, as highlighted in Figure 3 and in Scheme 2, the peaks due to the quaternary carbons C1 which resonate at 159.2 ppm, 159.25 and 159.03 ppm were assigned to the triads E-S-E, E-S-DPA and DPA-S-E, respectively. C_8_ peaks at 161.25 ppm were assigned to the asymmetric triads E-S-DPA and DPA-S-E, whereas the C_8_ signals at 161.09 ppm were assigned to the symmetric triads DPA-S-DPA.

As explained in the experimental section, the chemical microstructure of the copolymers such as the molar composition (C_ESES_ and C_ESDPA_), the triads sequence distribution (f_E-S-E_, f_E-S-DPA_, f_DPA-S-E_, and f_DPA-S-DPA_), the probability (P_DPA-E_ and P_E-DPA_) of finding the E (or DPA) unit next to that of DPA (or E), the number-average sequential length (L_ESES_ and L_ESDPA_) of the repeat units (ESES and ESDPA) and the degree of randomness of the P(ESES-co-ESDPA) copolymers were calculated from ^13^C-NMR spectra, applying the Equations (6)–(16) and utilizing the intensity of the carbons C_1_ and C_8_ (Table 3). The calculated molar compositions are noted in Table 1, whereas the other chemical microstructural data are summarized in Table 3. The calculated molar composition agrees with the DHDPS/DPA molar ratio in the feed suggesting that both monomers show a similar reactivity in the aromatic nucleophilic substitution polymerization reaction with the DCDPS monomer. All copolymers exhibit a degree of randomness ranging from 0.80 to 0.89, indicating that essential random copolymers are synthesized independently of the DHDPS/DPA molar ratio in the feed. As expected, the number-average sequential length of the ESES (L_ESES_) unit decreases as decreases their molar fraction, whereas that of the ESDPA units (L_ESDPA_) increases as raises their molar amount in the copolymers. The probability (P_E-DPA_) of finding the E moiety next to the DPA moiety decreases as raises the molar amount of ESDPA repeat units in the copolymer, whereas complementary and P_E-DPA_ increases. Beside the fundamental microstructural information, the study of ^13^C-NMR spectra also permits to identify the end groups of the copolymer chains. The signals at about 115.9 ppm and 114.7 ppm were assigned to the aromatic quaternary carbons linked to hydroxyl groups belonging the DHDPS (C_2′_) and DPA end groups (C_10′_), respectively. Moreover, the low intensity peaks at about 129.65 ppm were assigned to the aromatic tertiary carbons in meta position to the chlorine ends.

### 3.3. MALDI-TOF MS

**Figure 4:**
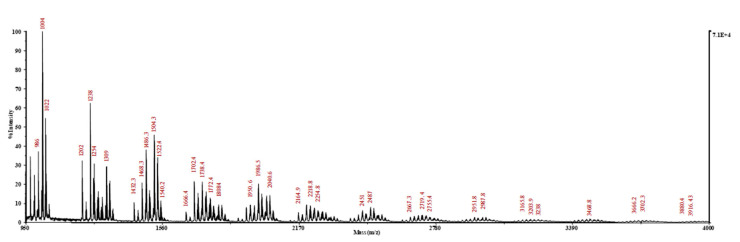
MALDI-TOF mass spectrum of the P(ESES-co-ESDPA) 50:50 copolymer.

**Figure 5:**
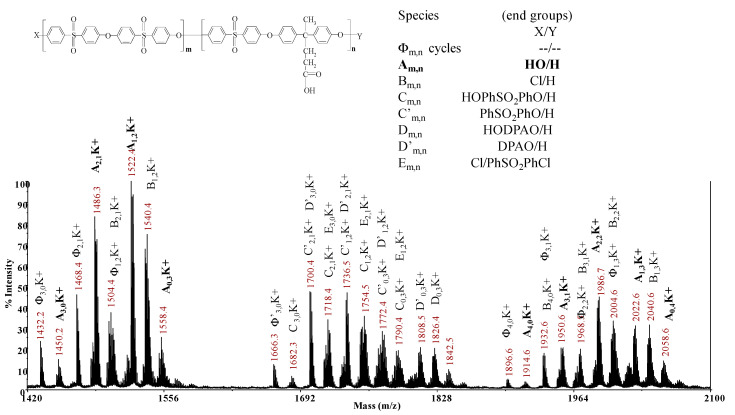
Enlarged section of the MALDI-TOF mass spectrum of the P(ESES-co-ESDPA) 50:50 copolymer.

All P(ESES-co-ESDPA) copolymers were also characterized by MALDI–TOF mass spectra, to obtain detailed information about their chemical structure. This powerful technique is able to look at the mass of individual molecules in a mixture of homologues, permitting the structural identification of the single macromolecular chain (linear and cycles). Often, MALDI-TOF mass spectra allow the identification of the repeat units, the end chains, cyclic oligomers and species present in minor amount [8,42,43,44,45,46,47,48]. MALDI-TOF mass spectra of all samples recorded in reflectron mode showed highly resolved mass peaks in the mass range *m/z* 1000–4000, permitting reliable and pertinent assignments to the corresponding (ESES)_X_-(ESDPA)_y_ co-oligomers. As an example, in Figure 4 is reported the mass spectrum of the copolymer P(ESES-co-ESDPA) 50:50, recorded in reflectron mode using DCTB as a matrix. It shows a series of homologous peaks in the mass range between *m/z* 950 and *m/z* 4000. In the expanded mass spectrum showed in Figure 5, are present intense peaks due to the potassium adduct ions of linear copolymer chains terminated with phenol groups at both ends (species A_m,n_, C_m,n_ and D_m,n_), to the copolymer chains terminated with OH at one end and with Cl at the other one (Species B_m,n_) and species terminated with chlorine at both ends (species E_m,n_). Co-oligomers terminated with mono hydroxy phenylsulfone (species C′_m,n_) or with mono hydroxy DPA (species D′_m,n_) were also observed. These unexpected end groups derive from the low amount (<1%) of the corresponding phenol present as impurity in the DHDPS, and DPA monomers, respectively. All copolymers synthesized exhibit the same end groups revealed by MALDI-TOF MS analysis of the P(ESES-co-ESDPA) 50:50. Moreover, beside to the potassium adduct ions of the linear co-oligomers, the mass spectra reveal the presence of the cyclic copolymer chains (species Φ_m,n_). The cyclic co-oligomers were not revealed by (^1^H and ^13^CNMR) analysis since they show chemical shifts very similar to those of the linear chains. Cycles were identified in the MALDI spectra of all copolymers studied and also in the corresponding homopolymers.

Therefore, further information about the structure of the copolymers studied is defined by MALDI-TOF MS analysis.

### 3.4. Size Exclusion Chromatography

**Figure 6:**
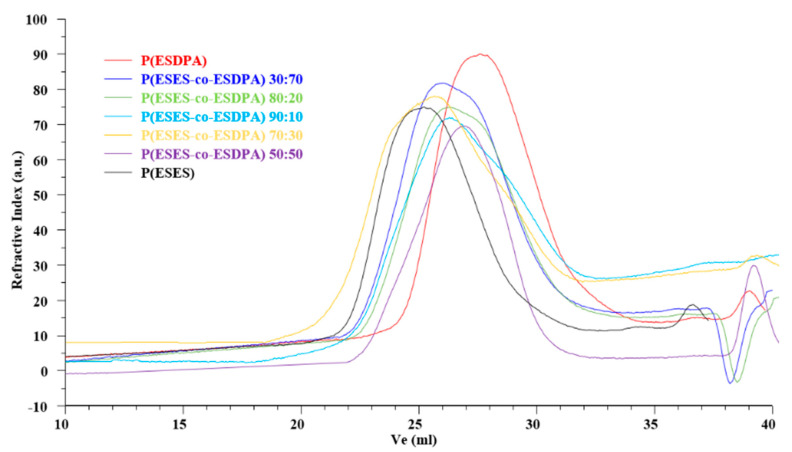
Overlay of the SEC traces of the P(ESES-co-ESDPA) copolymers and of their homopolymers, recorded using DMF as eluent.

In order to compare the molecular weights of the synthesized polymers, they have been analysed by size exclusion chromatography, using DMF as eluent and applying the calibration curve built with PS narrow polymer standards. Figure 6 shows SEC chromatograms of all polymers in the elution volume ranging from 10 mL up to 40 mL. The calculated average molar masses (Mw and Mn) are reported in Table 1. Either the P(ESES) homopolymer and the P(ESES-co-ESDPA) 70:30 copolymer shows average molar masses higher than those of other polymers. The P(ESDPA) homopolymer exhibits average molar mass lower than that of the other samples. All samples show polydispersity index (Mw/Mn) of about 2.1/2.2. The Mn values calculated by SEC analysis agree with those determined by ^1^H-NMR method (Table 1). P(ESES-co-ESDPA) copolymers 70:30, 50:50 and 30:70 were also analysed using THF as eluent and the chromatograms are displayed in Figure S7. The 70:30 sample has a lower elution time due to its slightly higher molecular weight, as confirmed by ^1^H-NMR analysis.

### 3.5. DSC

**Figure 7:**
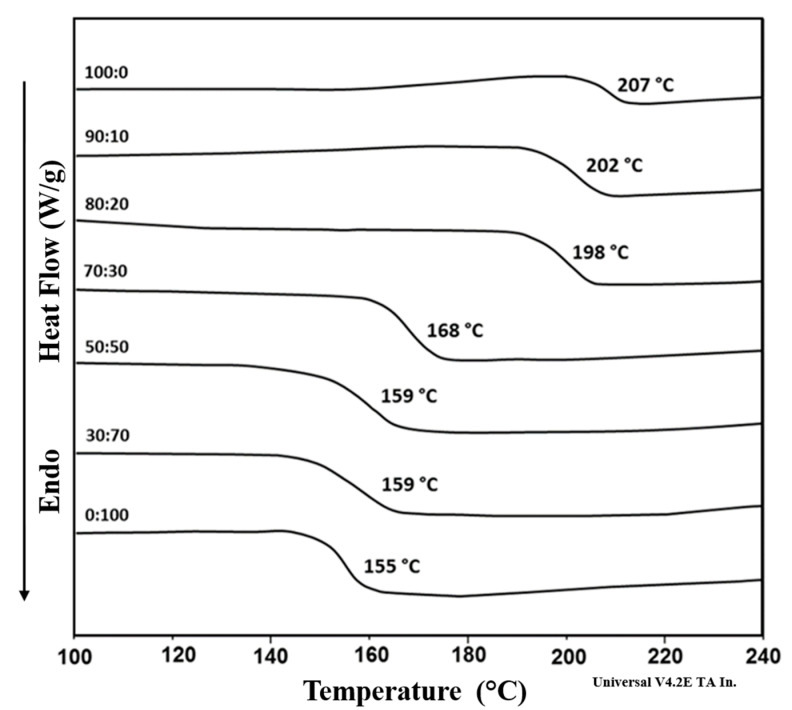
Thermograms of the second heating cycle of P(ESES-co-ESDPA) copolymers.

The thermal properties of all polymer samples were studied by DSC. All samples show only one glass transition temperature (Tg), and this behavior indicate that are amorphous materials. The Tg values are noted in Table 1. The P(ESES) homopolymer shows a Tg of 207 °C, very similar to that of commercial polymers (about 210 °C), whereas the P(ESDPA) homopolymer presents a Tg of 155 °C. These data suggest that the diphenolic unit influences the rigidity of the P(ESDPA) based polymers since the pendant COOH groups lead to a worsen packing of the chains. Moreover, the pentanoic acid linked to two phenyl moieties leads to a less rigidity of the DPA units with respect to the diphenylsulfone one, hampering the packing of the macromolecular chains. These effects lead to lowering of the Tg values of the P(ESDPA)copolymers with respect to the alternating poly(arylene ether-sulfone) (PES). The influence of the molar amount of ESDPA units on the Tg values was also observed in the P(ESES-co-ESDPA) studied. The Tg of the copolymers decreases as raises the molar amount of the ESDPA units in the chains, as displayed in Figure 7, showing the thermograms of the second heating cycle of P(ESES-co-ESDPA) copolymers. Comparing the data of the Tg values (Table 1) with those of number-average sequential lengths (L_ESES_) of ESES repeat units (Table 3) we deduced that the effect due to the DPA units prevails for L_ESES_ less of 4. The effect of pendant functional groups such as aldehyde moiety was already observed in modified polysulfones [30]. The authors suggest that this effect reduces the chain-stiffening on the polymer backbone.

### 3.6. DMA Analysis

**Figure 8:**
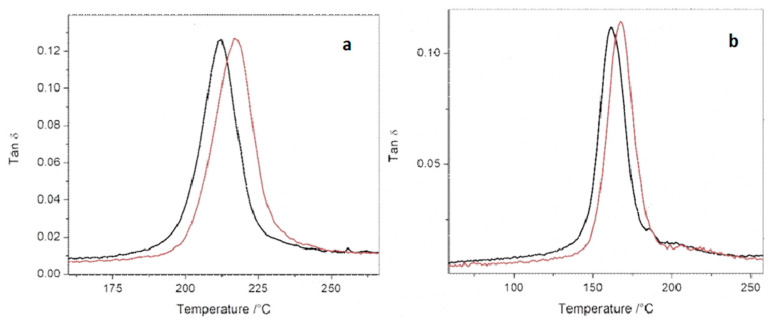
Tan δ versus temperature curves for (**a**) P(ESES) and (**b**) P(ESDPA) polymers: 1 Hz black line, 10 Hz red line.

**Figure 9:**
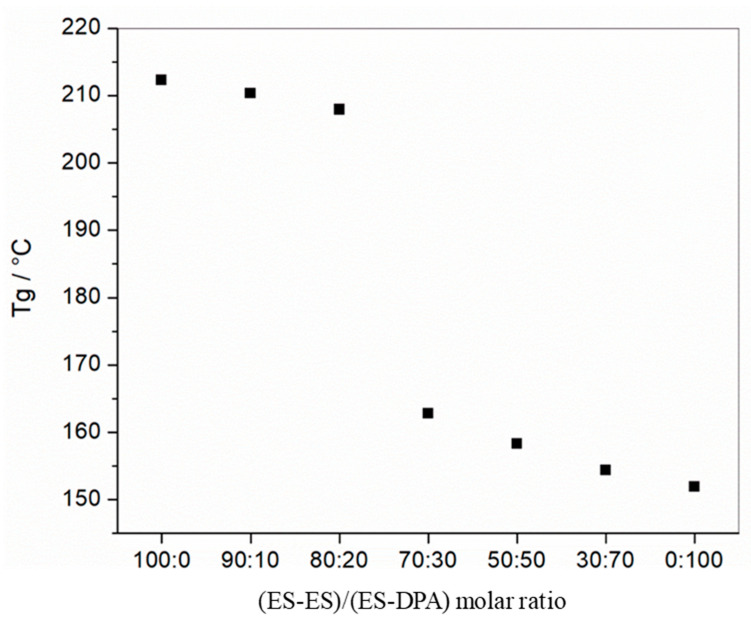
Glass transition temperature (Tg) of the copolymers versus the (ESES)/(ESDPA) molar ratio.

The thermomechanical properties of all samples were also investigated by DMA analysis. All the dried samples were tested under oscillatory mode while heating from 25 to 290 °C at 5 °C/min. Only the results of the second scan were considered. The DMA test was carried out in multifrequency mode to obtain the Tan δ traces at different frequencies. The results for P(ESES) and P(ESDPA) homopolymers are reported in Figure 8a,b. At 1 Hz a single peak was observed for both polymers at 212.3 °C and 160.5 °C for P(ESES), and P(ESDPA), respectively. The Tan δ shifted to 216.7 °C and 167.3 °C for P(ESES) and P(ESDPA) when measured at 10 Hz. The frequency shift of the Tan δ peak at high temperature is typically observed for glass transition relaxation phenomena. Similar behaviour was observed for all the copolymers tested. All the glass transition temperatures (Tg), measured as Tan δ peak at 1 Hz, for the copolymers with different molar composition of ES-DPA units, are plotted in Figure 9.

The glass transition temperature decreased with the increase of the molar amount of DPA units in the copolymer (Figure 8). The ESDPA units, bearing a flexible pendant group, increased the free volume through their effect on the package of the chains, with the consequent lowering of Tg.

**Scheme 3:**
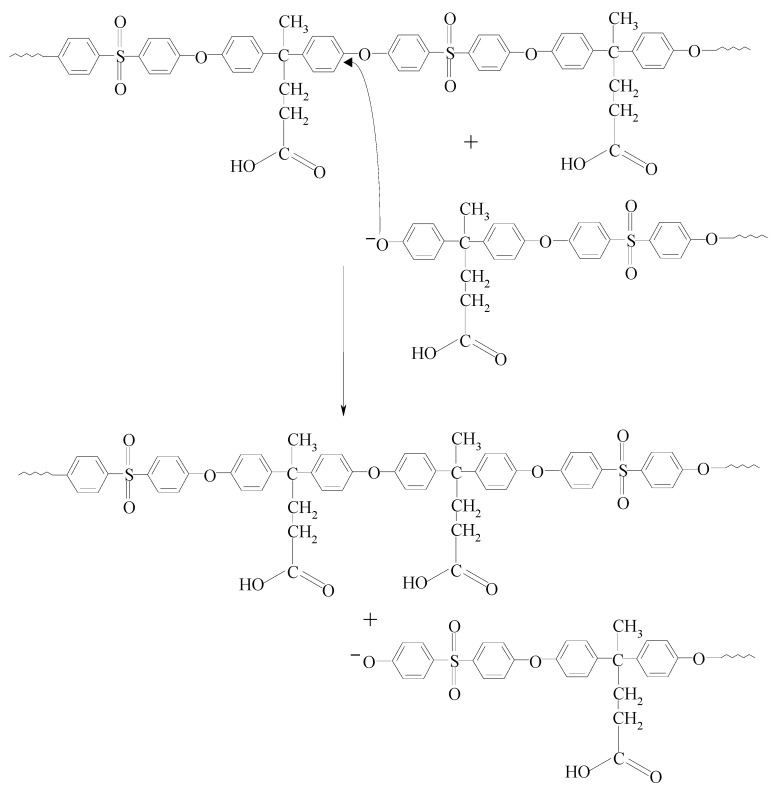
Trans-etherification reaction that generates the DPA-DPA homo-sequences along the P(ESES-co-ESDPA) copolymer chains, at temperature higher than 200 °C.

The trend of the Tg versus the mol% of the ESDPA molar units revealed a step Tg decrease for copolymer composition higher than 20% of ESDPA. This result can find its explanation in the formation of homogeneous DPA-DPA sequences (Phenyl-Alky-Phenyl-ether-Phenyl-alkyl-Phenyl-ether). These unexpected sequences can be generated by the trans-etherification reactions (Scheme 3) occurring at temperature higher than 200 °C, in accord to the DPMS data [24].

### 3.7. Wettability Analysis

The contact angle is an important parameter to characterize the hydrophilicity of a polymer sample. Its values indicate the hydrophobicity (>90°) and hydrophilicity (<90°), and the smaller the value, the better the hydrophilicity. The lacking of hydrophilic groups and the closing arrangement of macromolecular chains lead to the poor hydrophilicity for poly(arylene ether sulfone) (PES) Contact Angle values for each synthesized sample were determined, in order to compare the hydrophilicity of these new materials with the typical hydrophobicity of commercial PES. Each sample was measured 10 times and averaged. The experimental results are reported in Table 4.

**Figure 10:**
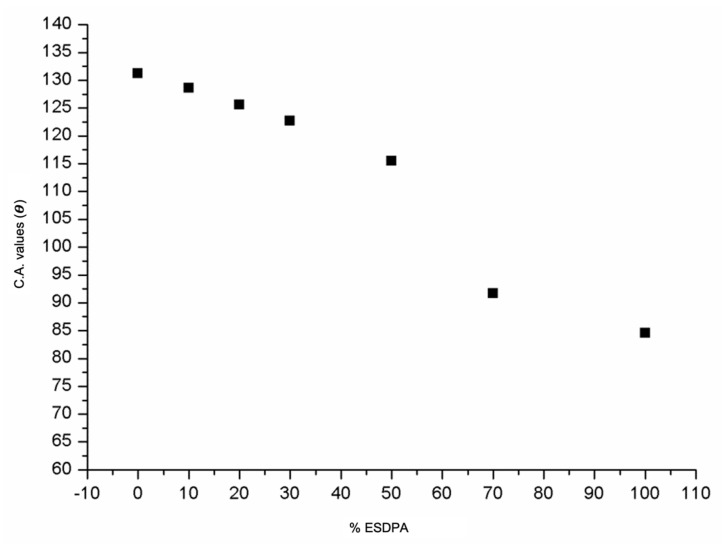
Contact Angle values (θ) vs. %ESDPA units.

Figure 10 shows the C.A. values plotted as function of the %ESDPA units. The P(ESES-co-ESDPA) 100:0 shows a value slightly lower than that of the corresponding commercial PES. This may be due to the lower average molar mass and a more concentration of OH end groups as revealed by ^1^H-NMR and MALDI-TOF MS analyses. Figure 10 and Table 4 show that as increases the molar amount of the DPA units in the copolymers the contact angle is reduced from 131.2° to 84.5°, and the polymer surface change from hydrophobic to hydrophilic. As expected, the P(ESDPA) homopolymer has the higher hydrophilicity (84.5°).

### 3.8. Selective Extraction on Heavy Metals

The application of commercial PES-based materials for the capture of heavy metals is described in literature [23,24]. In the present work, we report the results of a preliminary study aimed to test the selectivity of a (PESES-co-ESDPA) 50:50 membrane towards some cations of heavy metals, such as to chromium, vanadium, cadmium, arsenic, mercury, copper, zinc, iron, lead and cobalt. These metals if present in the environment, i.e., in the water, at levels above certain thresholds (generally in the order of ppm), can represent a serious hazard for human health. Water solutions with known concentrations (50 ppm) of chromium, cadmium, zinc and lead metals and their binary mixtures have been prepared. *Micro*- and *nano*-fibrous membranes were prepared by electrospinning of the (PESES-co-ESDPA) 50:50 copolymer synthetized. The recovery test was carried out by dipping the membranes for times ranging from 5 min to 7 days. The solutions, after the dipping times, were suitably diluted and characterized by means of a Nexion 300X ICP/MS spectrometer (Perkin Elmer Inc. Waltham, MA, USA); the difference between the initial and final concentration of the specific metal was thus assessed. The best results were obtained towards lead already after 5 min of dipping. A plateau was reached for immersion times higher than 10 min, due to the continuous exchange of metals between membranes and solutions. On the basis of these results, further studies will be carried out to improve the hydrophilicity of the membranes, by further specific functionalization of the (PESES-co-ESDPA) copolymers, and to extend the range of either extraction capability of several metal species and filtering power of the membranes.

## 4. Conclusions

By (^1^H and ^13^C)-NMR analysis the molar composition and the end chains of the P(ESES-co-ESDPA) copolymers synthesized were determined. The molar composition agrees with the molar ratio of both diphenol monomers (DHDPS and DPA) in the feed, suggesting that they have similar reactivity in the aromatic nucleophilic substitution polymerization reaction with the DCDPS monomer. ^13^C-NMR studies indicate that all polymer samples are essentially terminated with hydroxyl (OH) and/or Chlorine (Cl) moieties, as confirmed by MALDI-TOF MS analysis, which also reveals the formation of cycles both in the copolymers and in the homopolymers. The chemical microstructural analysis was carried out by ^13^C-NMR studies, applying appropriate equations and using the intensity of the aromatic quaternary carbons linked to the oxygen of the ether groups belonging to the sequences ESES and ESDPA. It reveals that essential random copolymers with a degree of randomness ranging from 0.80 up to 0.89, were synthesized. The number-average sequential length of the ESES units decreases as decreases their molar fraction, whereas that of the ESDPA units increases as raises their molar amount in the copolymers. Moreover, DSC and DMA studies suggest that the DPA units influence the thermal properties of the P(ESES-co-ESDPA) copolymers, since the Tg decreases with respect to that of (PESES) homopolymers as raises the ESDPA molar fraction. This unit influences also the hydrophilicity of the copolymers, since the contact angle values decrease as increase the ESDPA units in the copolymer chains. Furthermore, a preliminary study reveals that P(ES-co-ESDPA) 50:50 based membrane is efficient to extract lead ions from water solutions, in the presence of other heavy metals.

This work proves the feasibility of the proposed synthesis as a useful mean to obtain controlled structure for the P(ESES-co-ESDPA) copolymers. The present studies could be particularly useful to study the effect of the macromolecular structure on the properties of their parent/related membranes.

## Figures and Tables

**Table 1:** Some properties of the Poly(ether sulfone)s synthesized.

Sample	Feed Ratio (Mol Fraction)			Molar Composition (%_mol_) ^1^ ESES/ESDPA		Mn (g/mol) ^2^ (^1^H-NMR)	η_inh_^3^	Tg (°C)	PDI (Mw/Mn) ^4^	Yield ^5^ (%)
	DCDPS	DHDPS	DPA	^1^H-NMR	^13^C-NMR					
**1**	1.0	1.0	0.0	100/0	100/0	7900	0.35	207	2.19 (18200/8300)	93.2
**2**	1.0	0.9	0.1	88/12	89/11	7000	0.34	202	2.10 (15000/7150)	94.1
**3**	1.0	0.8	0.2	77/23	82/18	7100	0.33	198	2.15 (15700/7300)	92.7
**4**	1.0	0.7	0.3	68/32	71/29	8800	0.35	168	2.20 (19800/9000)	92.5
**5**	1.0	0.5	0.5	51/49	51/49	6600	0.32	159	2.19 (15000/6850)	94.6
**6**	1.0	0.3	0.7	32/68	33/67	7200	0.36	159	2.13 (15800/7400)	93.4
**7**	1.0	0.0	1.0	0/100	0/100	6000	0.30	155	2.26 (13900/6150)	91.2

^1^ Calculated through (^1^H and ^13^C)-NMR analyses. ^2^ Average Molar Mass determined by ^1^H-NMR. ^3^ Inherent Viscosity (ln ηr/c) c = 0.5 g/dL. ^4^ Measured by SEC analysis, using DMF as eluent. ^5^ Calculated after purification.

**Table 2:** Assignments, chemical shifts and integrations of signals observed in the spectra of the whole set of synthesized homopolymers.

Sequence Structures	H/C	Chemical Shifts (ppm)	
		Proton	Carbon
**ESDPA** 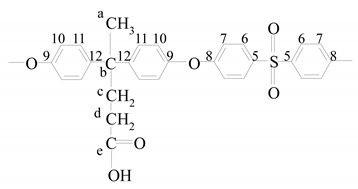	a	1.59 (s)	26.875
	b	-	44.5
	c	2.11 (t)	29.5
	d	2.38 (t)	35.9
	e	12.09 (bs)	173.88
	5	-	135.15
	6	7.91	128.5
	7	7.11	119.4
	8	-	161.91
	9	-	144.8
	10	7.26	117.7
	11	7.04	129.4
	12	-	152.3
**ESES** 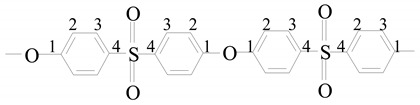	1	-	159.2
	2	7.26–7.29	119.63
	3	7.98–8.10	129.86
	4	-	136.6
**End groups** 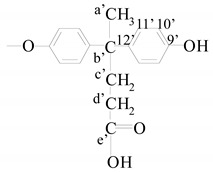	a′	1.53 (s)	25.48
	b′	-	44.1
	c′	1.88	30.2
	d′	2.18 (t)	36.6
	e′	12.07 (s)	172.93
	9′	-	145.4
	10′	6.69 (d)	114.7
	11′	6.95 (d)	130.44
	12′	-	153.05
	OH	9.12/9.19	-
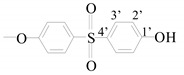	1′	-	162.12
	2′	6.82 (d)	105.8
	3′	7.70 (d)	129.45
	4′	-	134.4
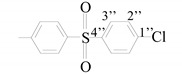	1″	-	139.33
	2″	7.69 (d)	129.04
	3″	7.92 (d)	129.62
	4″	-	138.78

**Table 3:** Normalized intensity of the aromatic quaternary carbon C1 and C8, molar fraction distribution of triads centred on the sulfone (S) moieties, probability of found E next to the DPA units and vice versa, number-average sequential lengths and degree of randomness of the copolymers studied.

Samples	Normalized Intensity of Carbons					Molar Fractions of Triads				P_E-DPA_	P_DPA-E_	L_ESES_	L_ESDPA_	β
	C8		C1											
	I_161.25_	I_161.09_	I_159.25_	I_159.2_	I_159.03_	f_E-S-E_	f_E-S-DPA_	f_DPA-S-E_	f_DPA-S-DPA_					
**80/20**	6.3	1.9	9.15	73.3	9.4	0.595	0.178	0.180	0.045	0.13	0.67	7.69	1.49	0.8
**70/30**	12.6	3.2	13.0	58.0	13.2	0.425	0.255	0.257	0.060	0.23	0.68	4.35	1.47	0.81
**50/50**	21.2	12.8	16.7	35.4	17.3	0.225	0.310	0.315	0.165	0.41	0.48	2.44	2.08	0.89
**30/70**	23.0	28.9	14.2	19.5	14.5	0.12	0.285	0.285	0.325	0.54	0.31	1.85	3.22	0.85

**Table 4:** Contact Angle values of the **P(ESES-co-ESDPA)** polymers and the commercial PES sample.

SAMPLE	AC (θ)
**P(ESES-co-ESDPA) 0:100**	84.5°
**P(ESES-co-ESDPA) 30:70**	91.7°
**P(ESES-co-ESDPA) 50:50**	115.5°
**P(ESES-co-ESDPA) 70:30**	122.7°
**P(ESES-co-ESDPA) 80:20**	125.6°
**P(ESES-co-ESDPA) 90:10**	128.6°
**P(ESES-co-ESDPA) 100:0**	131.2°
**Commercial PES**	137.7°

## Supplementary Materials

The following are available online at https://www.mdpi.com/2073-4360/12/8/1817/s1.
