# Supplementary material for: Synthesis and Characterization of Copoly(Ether Sulfone)s with Different Percentages of Diphenolic Acid Units
Source: Polymers (Basel). 2020 Aug 13;12(8):1817. doi: 10.3390/polym12081817 (PMC7465425; doi:10.3390/polym12081817)
Supplement: Supplementary file 1 [file polymers-12-01817-s001.zip › Figure Captions_Synthesis and Characterization of copoly(ether sulfone)s with different percentages of diphenolic acid units.docx]

## Synthesis and Characterization of copoly(ether sulfone)s with different percentages of diphenolic acid units

## Andrea A. Scamporrino^1)*^, Concetto Puglisi^1)^, Angela Spina^1)^, Maurizio Montaudo^1)^, Daniela C. Zampino^1)^, Gianluca Cicala^2)^, Giulia Ognibene^2)^, Chiara Di Mauro^2)^ Sandro Dattilo^1)^, Emanuele Mirabella^1)^, Giuseppe Recca^1)^, Filippo Samperi^1)^.

**Figure Captions:**

**Figure 1.** FTIR spectrum of the P(ESES-co-ESDPA) 30:70 copolymer.

**Figure 2.** ^1^H-NMR spectra in the aromatic region between 6.4 and 8.4 ppm of five polymer samples.

**Figure 3.** Enlarged section of seven ranges with assignments of the described spectra.

**Figure 4.** MALDI-TOF mass spectrum of the P(ESES-co-ESDPA) 50:50 copolymer.

**Figure 5.** Enlarged section of the MALDI-TOF mass spectrum of the P(ESES-co-ESDPA) 50:50 copolymer.

**Figure 6.** Overlay of the SEC traces of the P(ESES-co-ESDPA) copolymers and of their homopolymers, recorded using DMF as eluent.

**Figure 7**. Thermograms of the second heating cycle of P(ESES-co-ESDPA) copolymers.

**Figure 8.** Tan δ versus temperature curves for a) P(ESES) and b) P(ESDPA) polymers: 1 Hz black line, 10 Hz red line.

**Figure 9.** Glass transition temperature (Tg) of the copolymers versus the (ESES)/(ESDPA) molar ratio.

**Figure 10.** Contact Angle values (θ) vs %ESDPA units.
