# Supplementary material for: Synthesis and Characterization of Copoly(Ether Sulfone)s with Different Percentages of Diphenolic Acid Units
Source: Polymers (Basel). 2020 Aug 13;12(8):1817. doi: 10.3390/polym12081817 (PMC7465425; doi:10.3390/polym12081817)
Supplement: Supplementary file 1 [file polymers-12-01817-s001.zip › SI_Synthesis and Characterization of copoly(ether sulfone)s.docx]

## Synthesis and Characterization of copoly(ether sulfone)s with different percentages of diphenolic acid units

Andrea A. Scamporrino^1)*^, Concetto Puglisi^1)^, Angela Spina^1)^, Maurizio Montaudo^1)^, Daniela C. Zampino^1)^, Gianluca Cicala^2)^, Giulia Ognibene^2)^, Chiara Di Mauro^2)^, Sandro Dattilo^1)^, Emanuele F. Mirabella^1)^, Giuseppe Recca^1)^ and Filippo Samperi^1)^.

1) Institute for Polymers Composites and Biomaterials, IPCB-SS Catania CNR. Via Paolo Gaifami, 18 95126 Catania, Italy.

2) University of Catania, Department of Civil Engineering and Architecture, Viale Andrea Doria 6, 95125 Catania, Italy.

* Corresponding Author: Andrea A. Scamporrino

Email: [*andreaantonio.scamporrino@cnr.it*](mailto:andreaantonio.scamporrino@cnr.it)

IPCB-SS Catania CNR

Via Paolo Gaifami 18

95126 Catania, Italy

**Supporting Information**

**
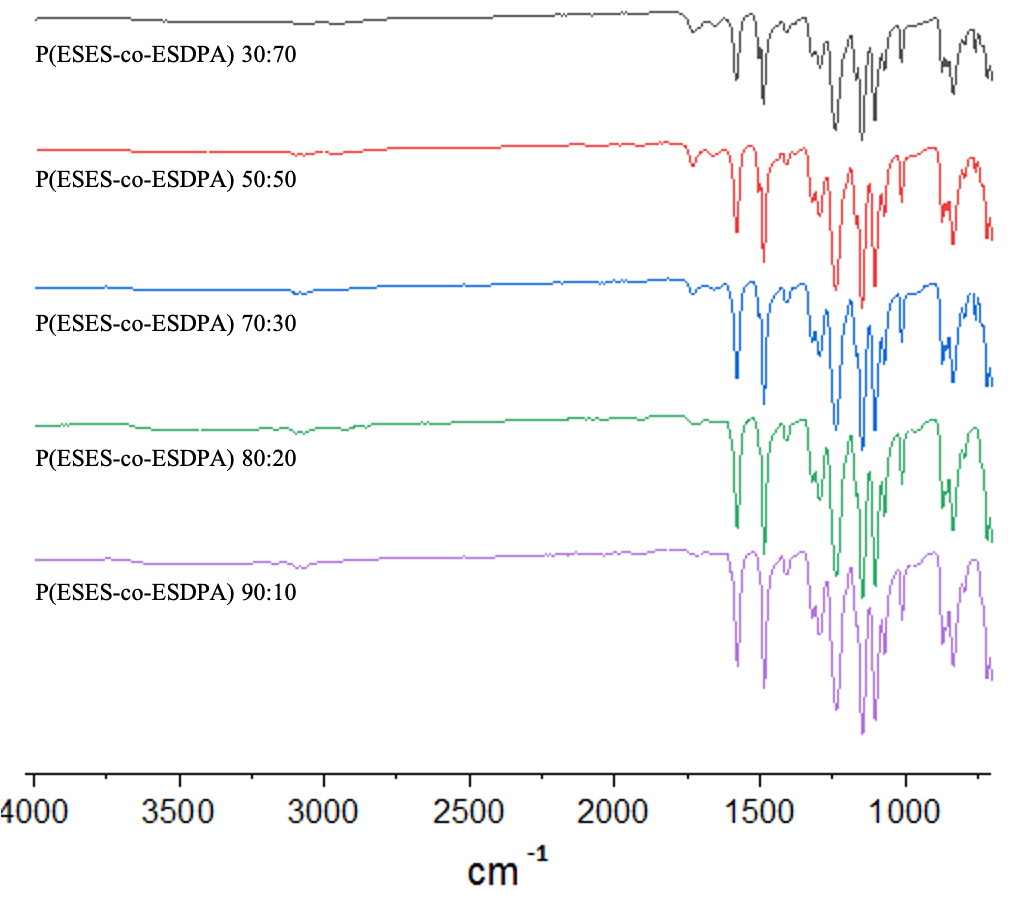
**

**Figure S1.** FT-IR spectra of P(ESES-co-ESDPA) copolymers.

- 1.
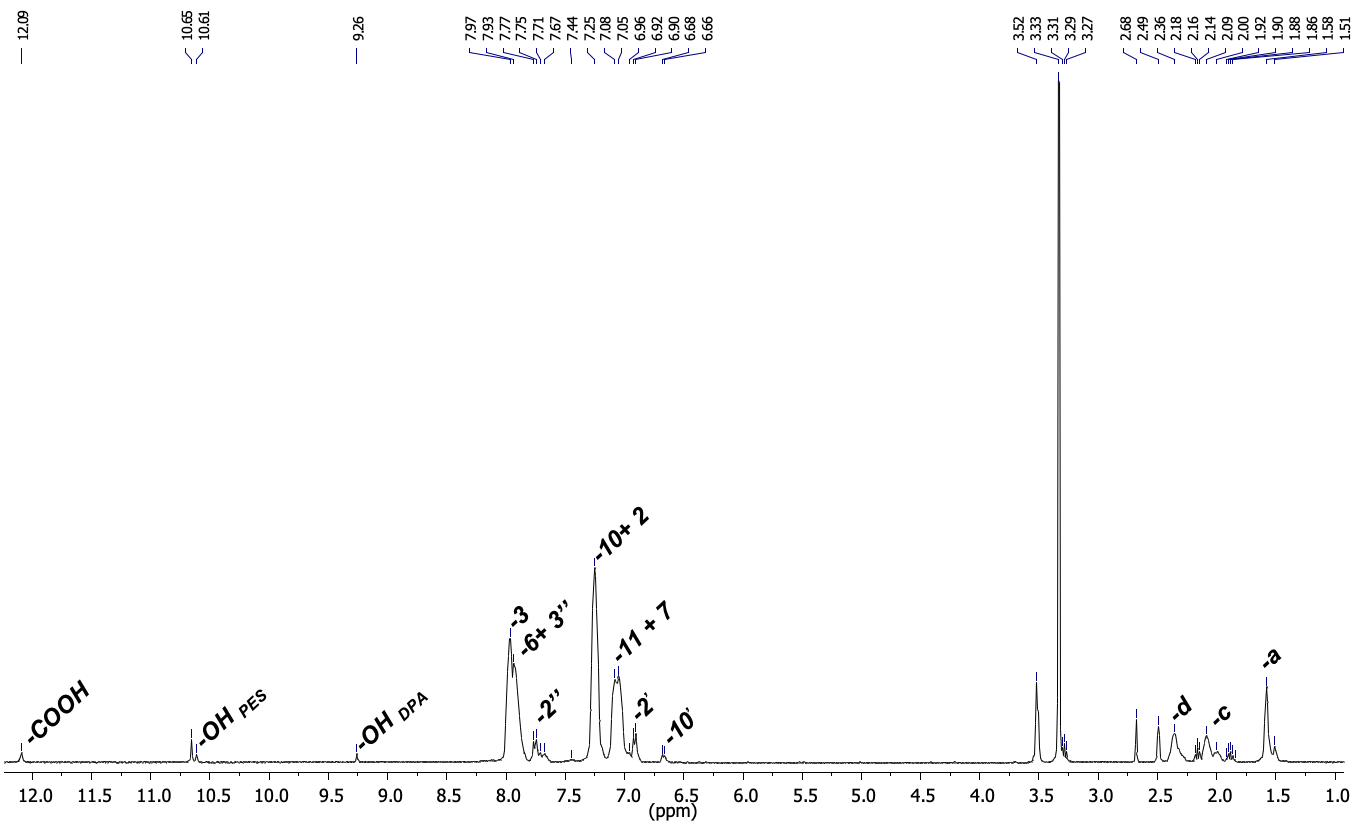
**^1^H-NMR**

**Figure S2.** ^1^H-NMR **(a)** and ^13^C-NMR **(b)** spectra of P(ESES-co-ESDPA) 50:50


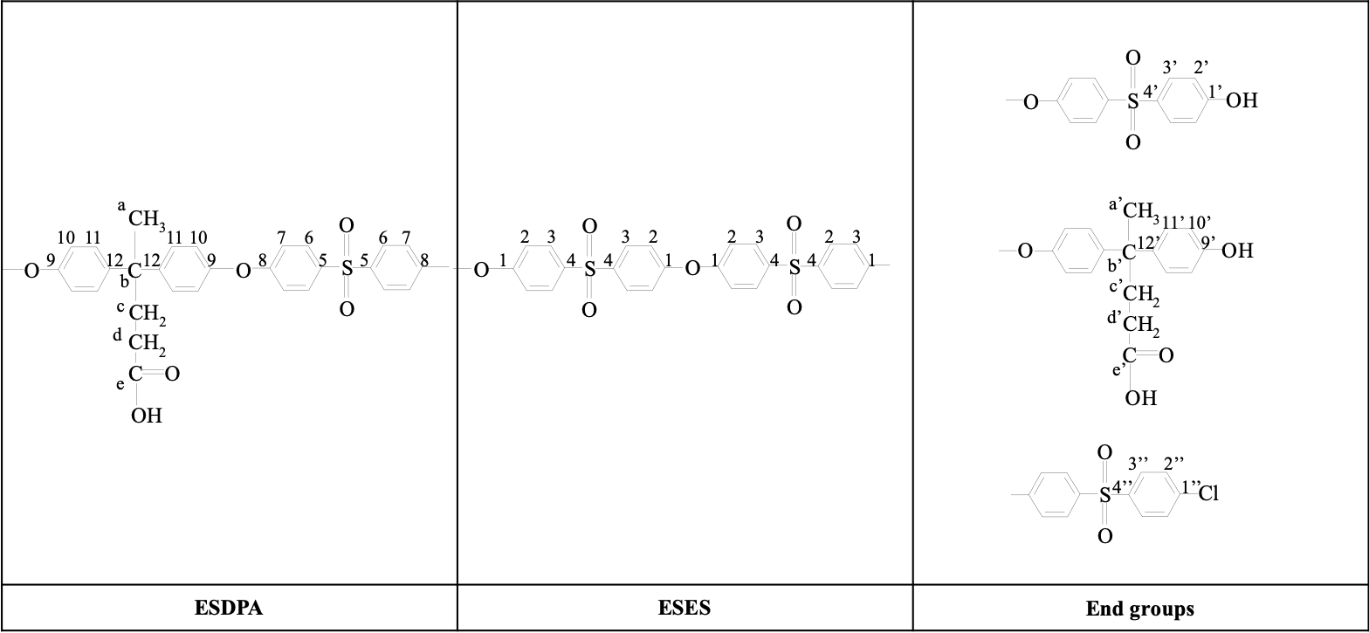


**Figure S3.** Proton and Carbon assignments of P(ESES-co-ESDPA) 50:50.

**Figure S4.** Aliphatic region of ^13^C-NMR spectra of P(ESES-co-ESDPA) copolymers: (a) 70.30, (b) 50:50 and (c) 30:70.

**Figure S5.** ^13^C-NMR spectrum of P(ESES) homopolymer.

**Figure S6.** ^13^C-NMR spectrum of P(ESDPA) homopolymer.

**
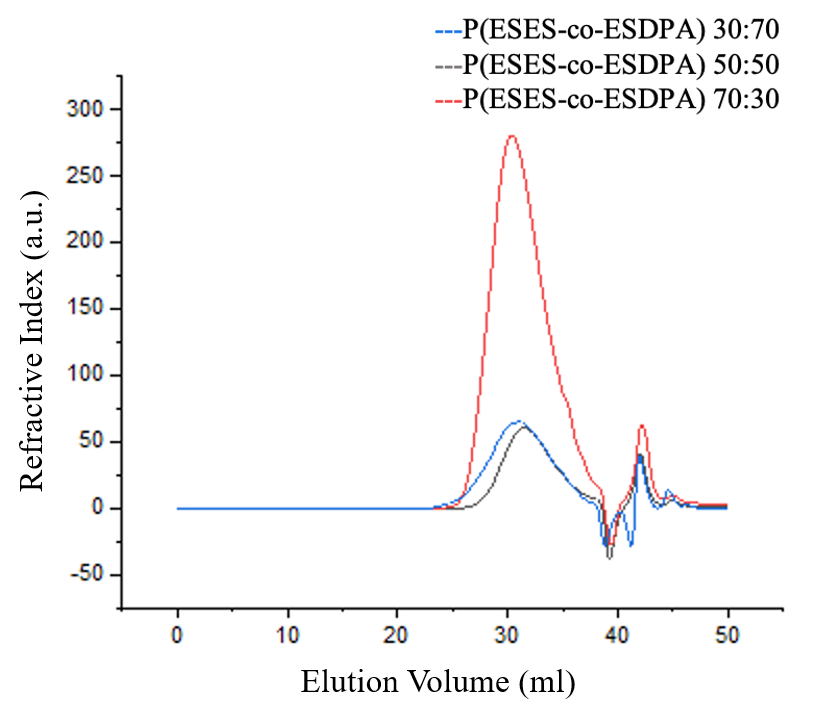
**

**Figure S7.** Overlay of the SEC traces of three P(ESES-co-ESDPA) samples 30:70, 50:50 and 70:30 recorded using THF as solvent.
